# Transgenic mouse models expressing human and macaque prion protein exhibit similar prion susceptibility on a strain-dependent manner

**DOI:** 10.1038/s41598-019-52155-z

**Published:** 2019-10-30

**Authors:** Juan Carlos Espinosa, Emmanuel E. Comoy, Alba Marin-Moreno, Patricia Aguilar-Calvo, Marie-Christine Birling, José Luis Pitarch, Jean-Philippe Deslys, Juan María Torres

**Affiliations:** 1 Centro de Investigación en Sanidad Animal (INIA-CISA), 28130 Valdeolmos, Madrid Spain; 2 CEA, Institut François Jacob, Université Paris-Saclay, 18 Route du Panorama, 92265 Fontenay-aux-Roses, France; 3 Institut Clinique de la Souris, 1 rue Laurent Fries, BP 10142, 67404 Illkirch, France

**Keywords:** Animal disease models, Animal disease models, Molecular neuroscience, Molecular neuroscience

## Abstract

Cynomolgus macaque has been used for the evaluation of the zoonotic potential of prion diseases, especially for classical-Bovine Spongiform Encephalopathy (classical-BSE) infectious agent. PrP amino acid sequence is considered to play a key role in the susceptibility to prion strains and only one amino acid change may alter this susceptibility. Macaque and human-PrP sequences have only nine amino acid differences, but the effect of these amino acid changes in the susceptibility to dissimilar prion strains is unknown. In this work, the transmissibility of a panel of different prions from several species was compared in transgenic mice expressing either macaque-PrP^C^ (TgMac) or human-PrP^C^ (Hu-Tg340). Similarities in the transmissibility of most prion strains were observed suggesting that macaque is an adequate model for the evaluation of human susceptibility to most of the prion strains tested. Interestingly, TgMac were more susceptible to classical-BSE strain infection than Hu-Tg340. This differential susceptibility to classical-BSE transmission should be taken into account for the interpretation of the results obtained in macaques. It could notably explain why the macaque model turned out to be so efficient (worst case model) until now to model human situation towards classical-BSE despite the limited number of animals inoculated in the laboratory experiments.

## Introduction

Transmissible Spongiform Encephalopathies (TSEs) are a group of fatal neurodegenerative diseases, which include Bovine Spongiform Encephalopathy (BSE) in cattle, scrapie in sheep and goats, Chronic Wasting Disease (CWD) in cervids and Creutzfeldt-Jakob disease (CJD) in humans. It is considered that the causative agent of TSEs consists mainly, if not entirely, of PrP^Sc^ which is an abnormal isoform of the host encoded prion cellular protein, PrP^C^ ^1–4^. TSEs are also called prion diseases. Prion propagation is considered to occur in a two-step process where aggregates of PrP^Sc^ interact with PrP^C^ and then induce their conformational change into PrP^Sc^. The conversion in PrP^Sc^ results in changes in physical properties as an increase in β-pleated sheet content and resistance to proteolytic degradation and decreased solubility in non-denaturing detergents.

Upon experimental transmission, TSE strains show distinctive biological features (such as incubation periods and neuropathology) which are preserved after sub-passage in the same species. According to the prion hypothesis^5^, different PrP^Sc^ conformations are associated with dissimilar strains. Prion transmission barrier among different species seems to be driven by the differences in the PrP amino acid sequences and by the prion strain being transmitted^6,7^. While PrP amino acid sequence seems to play a key role in prion transmission susceptibility, host factors other than PrP^C^ can modulate prion strain features^7,8^. Transgenic mouse models null for murine *Prnp* gene and expressing the PrP^C^ from another species (such as bovine, ovine, porcine, human, etc), have been used in the prion research field as useful tools to characterise prion strains and to learn about the transmissibility of prion strains to different species^6,7,9^, and in particular the susceptibility of humans to prions^10–16^.

**Figure 1:**
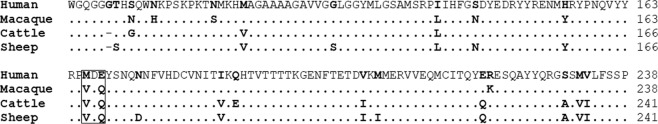
Amino acid comparison of human macaque, cattle and sheep PrP amino acid sequences. Only amino acids 89 to 238 (according to human PrP) are included in the comparison for clarity. Points indicate identical residues. Deletions are indicated by dashes. Amino acid numbering is indicated on the right. Species are named on the left. Amino acid changes in 166 and 168 positions (M/V and E/Q respectively) are boxed.

Several studies have been done using non-human primates to study the transmissibility of prion diseases^17,18^ and more recently, macaque monkeys have been widely used for prion disease transmissions^19–30^. In this sense, non-human primates are considered to be the ultimate model of the human condition with regard to prions, especially for BSE infection^19,22^. Both macaque and human PrP amino acid sequences are quite similar, but only one amino acid change may alter susceptibility to prions drastically, as occurs with the Met/Val 129 dimorphism in human PrP sequence for classical-BSE prion strain^14^. The nine amino acid differences between human and macaque PrP (see Fig. 1) may alter prion susceptibility of these two species. In this work, we address this question comparing the susceptibility of transgenic mouse models expressing either human or macaque PrP when inoculated with a panel of diverse prions.

## Results

### Macaque PrP^C^ expression in transgenic mice

**Figure 2:**
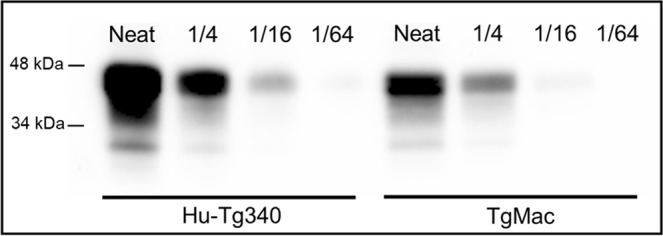
Brain PrP^C^ expression in TgMac mouse line in comparison to Hu-Tg340 brain. Immunoblots of the brain PrP^C^ expression detected with 12B2 mAb. Direct sample (10% brain homogenates) and ¼ dilutions were loaded on 12% Bis-Tris gels.

PrP^C^ expression in brain from homozygous TgMac mice was checked by W estern blot using a specific anti-PrP monoclonal antibody (12B2). Brain PrP^C^ expression levels for the TgMac mice were found to be around half than the PrP^C^ levels found in Hu-Tg340 brains. PrP^C^ from TgMac mice showed a similar electrophoretic profile than the PrP^C^ obtained from the brain of Hu-Tg340 mice (Fig. 2). Neither behavioural defects such as neurological signs, social deficits or alterations in reproduction rates, nor reduction in their lifespan were observed in TgMac mice.

### Comparison of prion infection susceptibility in TgMac and Hu-Tg340 mice

TgMac and Hu-Tg340 mice were inoculated through the intracerebral route with a collection of isolates representative of different prion strains (Table 1) from human, sheep and cattle. The susceptibility to prion infection of both mouse lines expressing either human or macaque PrP^C^ was compared using the same inocula.

**Table 1:** Description of the isolates used in this study.

Isolate	Description and references	Supplier
sCJD 129 M/M T1	Type 1 sCJD M129M infected case (0.08.02523_001)	BB^a^
sCJD 129 V/V T2	Type 2 sCJD V129V infected case (0.08.02497_001)	BB
vCJD	vCJD M129M infected case (BC1458)^14^	BHUFA^b^
BSE	Naturally BSE infected cow^54^	AHVLA^c^
BSE H	Atypical H-BSE natural case from Poland. Po 45^48^	NVRI^d^
BSE L	Atypical L-BSE natural case from Poland. Po 15	NVRI
Sheep-Sc PS21	Naturally scrapie-infected sheep, ARQ/ARQ. Fr (21)^31^ from France	INRA^e^
Sheep-Sc198-9	Naturally scrapie-infected sheep, ARQ/ARQ. It (198-9) from Italy	ISS^f^

^a^Basque Biobank. Bilbao. Spain.

^b^Biobanco Hospital Universitario Fundación Alcorcón. Alcorcón. Spain.

^c^Animal Health and Veterinary Laboratories Agency. New Haw. Addlestone Surrey, UK.

^d^National Veterinary Research Institute. Pulawy. Poland.

^e^French National Institute for Agricultural Research, Nouzilly, France

^f^Instituto Superiore di Sanitá. Rome. Italy.

#### Transmission of human prions

**Figure 3:**
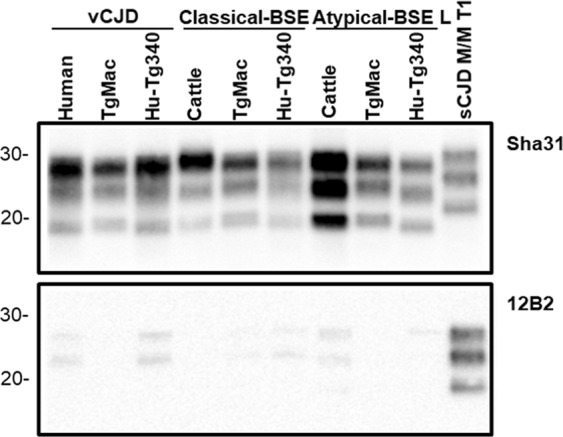
Biochemical comparison of brain PrP^res^ in TgMac or Hu-Tg340 mice. Electrophoresis profiles and antibody labeling of PrP^res^ as detected with mAbs Sha31 (top) and 12B2 (bottom) in brain extracts from TgMac or Hu-Tg340 mice infected with the prion agents indicated in the top. The original isolates either from human or cattle were also included for biochemical comparative purposes. Similar quantities of PrP^res^ were loaded in each lane for a better comparison. Brain extract from Human sCJD 129M/M T1 (sCJD T1) was included as positive control for 12B2 antibody labeling. Both panels were loaded with the same quantities of PrP^res^ extracted from each sample. Molecular weights (in kDa) are shown at the left of the blot.

**Figure 4:**
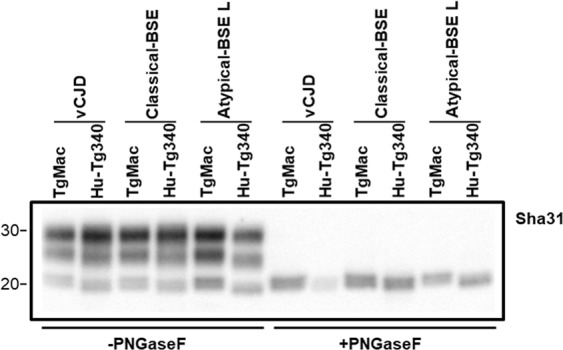
Biochemical comparison of brain PrP^res^ unglycosylated band in TgMac or Hu-Tg340 mice. Electrophoresis profiles of PrP^res^ as detected by mAb Sha31 in brain extracts from TgMac or Hu-Tg340 mice infected with the prion agents indicated in the top. Brain PrP^res^ were untreated (left panel) or treated (right panel) with PNGaseF. Molecular weights (in kDa) are shown at the left of the blot.

**Figure 5:**
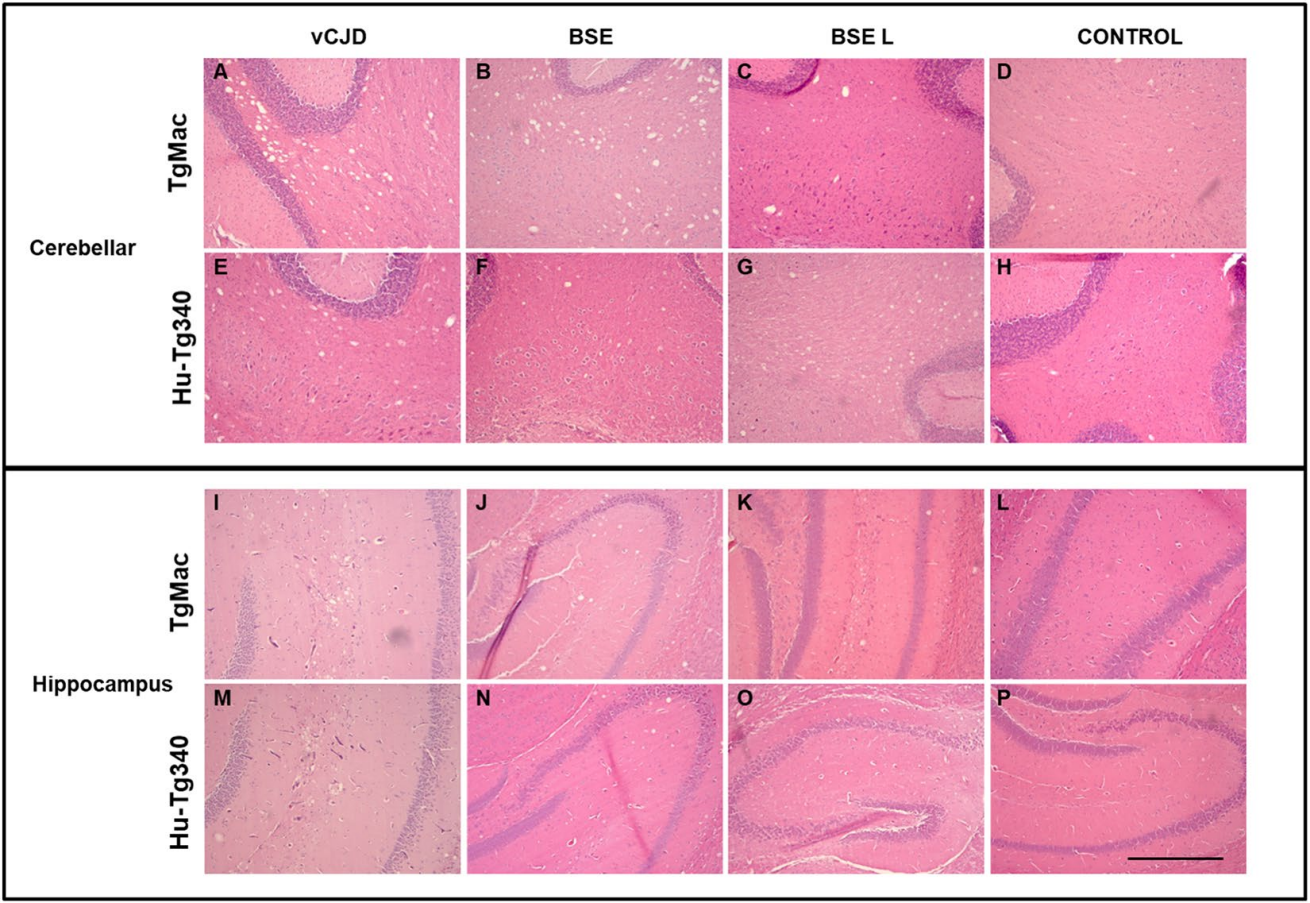
Comparative histopathology of vCJD, BSE and BSE L in TgMac and Hu-Tg340. Spongiosis in cerebellar white matter (**A**–**H**) or in the hippocampus (**I**–**P**) in both TgMac (**A**–**D** and **I**–**L**) and Hu-Tg340 (**E**–**H** and **M**–**P**) inoculated with vCJD, BSE, BSE L and non-inoculated control as indicated. Scale bar = 500 μm.

**Figure 6:**
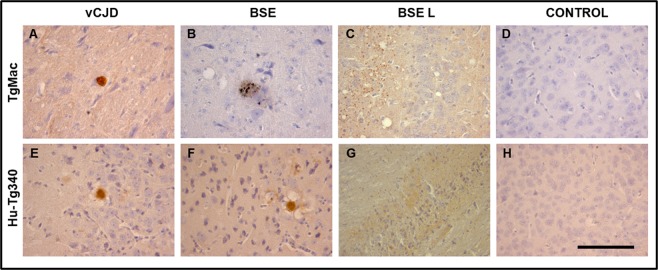
PrP^res^ immunostaining in brains from TgMac and Hu-Tg340 infected with vCJD, BSE or BSE L. All panels show the PrP deposition with Sha31 monoclonal antibody. Cerebral cortex in vCJD, BSE, and non-inoculated control TgMac (**A**,**B** and **D)** or Hu-Tg340 (**E**, **F** and **H**). Thalamus in BSE L inoculated animals (**C** and **G**). Immature florid plaque with a dense core of PrP in the cerebral cortex of TgMac inoculated with vCJD (**A**). Dense plaques in the cerebral cortex of TgMac inoculated with BSE (**B**). Florid plaques in the cerebral cortex of Hu-Tg340 inoculated with vCJD (**E**). Florid plaques in the cerebral cortex of Hu-Tg340 inoculated with BSE after second passage^54^ **(F**) are shown because in the first passage, with only one positive animal, no plaques were detected. PrP^res^ distributed in a diffuse pattern in the thalamus of TgMac (**C**) or Hu-Tg340 (**G**) inoculated with BSE L. No staining was observed in the brain of non-inoculated control mice in these conditions (**D** and **H**). Scale bar = 100 μm.

Infection with sCJD prion isolates was not detected in TgMac mice as none of them was scored positive for PrP^res^ after long survival times (Table 2). As expected, sCJD isolates infected efficiently Hu-Tg340 mice with 100% attack rates. By contrary, vCJD isolate transmitted to TgMac mice with 100% attack rates and with a mean survival time similar to the one obtained for Hu-Tg340 mice inoculated with vCJD (537 ± 105 vs 545 ± 146 dpi) (Table 2). Similar antibody labelling and brain-PrP^res^ glycoprofile with a predominant diglycosylated band was revealed by Western blot in both mouse lines for vCJD inoculum, but a very small reduction in the electrophoretic mobility of the macaque-PrP^res^ unglycosylated band was observed when compared to the human-PrP^res^ counterpart (Figs 3 and 4). The predicted molecular mass for macaque-PrP according to its amino acid sequence is slightly higher than for human-PrP (27676 Da and 27661 Da respectively). This minor difference in the molecular mass (15 Da) could explain the small difference observed in the electrophoretic mobility. A different proteinase K cleavage in the brain PrP^res^ from either TgMac or Hu-Tg340 mice seems unlikely as the antibody labelling is similar in all cases (Fig. 3). Neuropathological features (lesion profile and PrP^res^ distribution in Pet blot) observed in brains from vCJD inoculated TgMac mice were similar to those previously described for Hu-Tg340 mice inoculated with vCJD^14^ showing prominent vacuolation at the cerebellar and hippocampus level (Fig. 5). Immature forms of florid plaques were observed in the brains of TgMac mice inoculated with vCJD (Fig. 6) as previously described in macaques inoculated with vCJD^20^.

**Table 2:** Transmission of human, bovine and sheep isolates to TgMac and Hu-Tg340 mouse models.

Inocula	Mean survival time in days ± SD, (n/n_0_)^a^	
	TgMac	Hu-Tg340
sCJD 129 M/M T1	>650 (0/5)	185 ± 7 (7/7)
sCJD 129 V/V T2	>650 (0/5)	522 ± 36 (6/6)
vCJD	537 ± 105 (5/5)	545 ± 146 (5/5)^b^
BSE	518 ± 106 (7/7)	>650 (1/8)^c^
BSE H	>650 (0/5)	>650 (0/6)
BSE L	550 ± 86 (7/7)	607 ± 13 (4/4)
Sheep-Sc PS21	273–392 (2/6)	>650 (0/6)
Sheep-Sc198-9	>650 (0/6)	>650 (0/6)
Healthy cattle brain	>650 (0/6)	>650 (0/6)

^a^n/n_0_: diseased, PrP^res^ positive/inoculated animals. Survival times are indicated as mean ± SD dpi of all the mice scored positive for PrP^res^.

^b^Published in reference^14^.

^c^Published in reference^54^.

#### Transmission of cattle prions

Neither TgMac nor Hu-Tg340 mice were scored positive for the transmission of the disease when inoculated with atypical-BSE H. However, atypical-BSE L transmitted efficiently in both mouse lines (100% mice were scored positive; Table 2) showing similar long survival times (550 ± 86 vs 607 ± 13). Neuropathological features observed in brains from atypical-BSE L inoculated TgMac mice were similar to those observed in Hu-Tg340 mice inoculated with the same prion isolate (Figs 5 and 6). There were not significant differences in antibody labelling and the brain-PrP^res^ glycoprofile in Western blot in both mouse lines inoculated with atypical-BSE L (Fig. 3).

Remarkably, while only one out of eight of the Hu-Tg340 mice inoculated with classical-BSE was scored positive for the transmission of the disease, all the TgMac mice inoculated with classical-BSE were positive for brain PrP^res^ after 518 ± 106 dpi (Table 2). A similar antibody labelling and brain-PrP^res^ glycoprofile was observed in both mouse lines inoculated with classical-BSE (Fig. 3). This glycoprofile was also similar to that observed after inoculation of both mouse lines with vCJD. As previously observed in both lines inoculated with vCJD, a slight reduction in the electrophoretic mobility of the macaque-PrP^res^ unglycosylated band was observed when compared to the human-PrP^res^ counterpart after inoculation with classical-BSE or atypical-BSE L (Figs 3 and 4). Neuropathological features (lesion profile and PrP^res^ distribution in Pet blot) observed in brains from classical-BSE inoculated TgMac mice were similar to those previously reported for Hu-Tg340 mice after infection with classical-BSE^16^. Dense plaques representing immature forms of florid plaques were observed in the brains of TgMac mice inoculated with classical-BSE (Fig. 6) as previously described in macaques inoculated with classical-BSE^20^.

#### Transmission of sheep prions

In accordance with previous studies^31^, none of the Hu-Tg340 mice inoculated with scrapie was scored positive for the disease while two out of six TgMac mice were scored positive for the disease at 273 and 392 dpi when inoculated with Sheep-Sc PS21 scrapie isolate (Table 2). Both TgMac mice scored positive showed sparse PrP^res^ accumulation in their brain. None of the TgMac mice inoculated with Sheep-Sc198-9 was scored positive for the disease.

## Discussion

In this work, the transmission features of a collection of TSE isolates, representing a panel of diverse prion strains, is compared in two transgenic mouse experimental models expressing either macaque-PrP^C^ or human-PrP^C^. Human and macaque transmission barriers for different prions are compared in identical conditions in the same context. The main differences in both transgenic models are included in the PrP amino acid sequence expressed in each transgenic mouse line. The use of macaque species as a model for prion transmission in humans^32^ remarks the importance of a deep analysis of the differences in the prion transmission barrier of human versus macaque PrP amino acid sequences.

Results presented here showed a strain dependent susceptibility of both mouse models to the panel of inoculated prion isolates. Atypical-BSE showed similar transmission features in both Hu-Tg340 and TgMac, suggesting that the amino acid differences between macaque and human-PrP^C^ are irrelevant for the transmissibility of both atypical-BSE strains. The ability of atypical BSE-L prion strain to infect both transgenic mouse lines expressing human or macaque-PrP^C^ is in agreement with previous experiments published either in transgenic mice expressing human-PrP^C^ ^33–35^ or in macaques^23^. In parallel, the inability of atypical BSE-H prion strain to infect both transgenic mouse lines expressing human or macaque-PrP^C^ is in agreement with previous experiments in transgenic mice expressing human-PrP^C^ ^33^ or in macaques^28^.

By contrast, different transmission features were observed in Hu-Tg340 and TgMac inoculated with other prion strains such as classical-BSE. The susceptibility observed in mice expressing macaque PrP^C^ is higher than in those expressing human PrP^C^ in terms of attack rate. This information must be considered when macaque species is used for the evaluation of the transmissibility of animal prions to human species. It could notably explain why the macaque model turned out to be so efficient (worst case model) until now to model human situation towards BSE despite the limited number of animals inoculated in the laboratory experiments. In a similar way, Hu-Tg340 and TgMac mice show identical transmission features for vCJD (Table 2). This behavior beside the anatomical similarities of human and macaques supports the suitability of macaques as a valuable tool for modeling vCJD secondary transmissions in humans.

Table 3 shows a compilation of data from different experiments using macaque species for the evaluation of the transmissibility of different prions^21,23,28^. These data should be compared with caution, as there are experimental differences in the inocula used, inoculation route, etc. Anyway, in most cases, the data shows a comparable transmissibility in both macaque and transgenic mice expressing macaque PrP.

**Table 3:** Transmissions of human, bovine and sheep prions to macaques previously reported.

Inocula	Mean survival time in months (n/n_0_)^a^
sCJD 129 M/M T1	56, 62, 63 (3/3)^b^
sCJD 129 V/V T2	>170^c^
vCJD 129M/M	25, 30 (2/2)^d^
BSE	36, 40, 40 (3/3)^d^; 35, 37, 59 (3/3)^e^
BSE H	>122^f^; >163^c^
BSE L	26 (1/1)^g^; 24, 25 (2/2)^h^
Sheep-Scrapie	118 (1/1)^f^; 67 (1/1)^i^; 73 (1/1)^j^; 82 (0/1)^j^

^a^n/n_0_: diseased, PrP^res^ positive/inoculated animals.

^b^Published in reference^21^.

^c^Unpublished results.

^d^Published in reference^20^.

^e^Published in reference^25^.

^f^Published in reference^28^.

^g^Published in reference^23^.

^h^Published in reference^24^.

^i^Published in reference^18^.

^j^Published in reference^43^.

Macaque-PrP presents an important transmission barrier for human prion strains such as type 1 or type 2 sCJD as none of these strains was able to generate a detectable infection in transgenic mice expressing macaque-PrP within the time frame of these experiments. Previous data evidenced that macaques can be infected with type 1 sCJD showing incubation periods around twice than in the case of vCJD prion strain (56 months versus 25, 30, 32 and 37 months^21^). Given that the survival time observed for vCJD in mice expressing macaque-PrP is close to the end point of these experiments, the transmission barrier for sCJD would impede the detection of type 1 sCJD in the mouse model. When compared with human-PrP, macaque-PrP amino acid sequence presents the R to K change in 220 position (see Fig. 1). It is remarkable that 220 amino acid position is adjacent to the E219K polymorphism described in humans, which has been linked to protecting humans against sCJD in epidemiological studies in Asiatic populations^36,37^. E219K polymorphism has been associated to perturbations in surface change distribution and structural rearrangements (mainly localized at the β2-α2 loop region). Similarly, we can speculate that the R220K change observed in macaque-PrP would alter the indicated zone, providing a protective effect to macaques for sCJD infection. Moreover, when compared with human-PrP, macaque-PrP presents two amino acid changes at the 166 and 168 positions also present in cattle and sheep PrP sequences (M/V and E/Q respectively, see Fig. 1). Both 166 and 168 amino acid changes are in the β2-α2 loop of PrP. Changes in the β2-α2 loop are deeply related with alterations in the susceptibility to prion strains^38–41^. In particular, the amino acid changes observed in 166 and 168 positions are included in sequence elements implicated in both resistance to CWD in humans and in delaying the disease progression of sCJD^42^. The conservation of both V166 and Q168 amino acids in macaque, cattle and sheep PrP, three species highly susceptible to classical-BSE infection, allows as to propose these residues as determinant elements for the susceptibility to classical-BSE.

Finally, macaque PrP presents a differential susceptibility for several scrapie isolates, providing a tool discriminating scrapie prion strains. Previous studies in macaques report susceptibility to scrapie from 3 of 4 scrapie inocula (see Table 3) suggesting that different results may be obtained in animals expressing macaque-PrP due to differences in the inoculated scrapie strain^18,28,43^.

Overall, although both Tg-Mac and Hu-Tg340 mice showed a similar susceptibility to vCJD, Tg-Mac mice present an important transmission barrier to other human prion strains such as type 1 or type 2 sCJD suggesting a strain dependent susceptibility of Tg-Mac to human prions. On the other hand, the similar transmission properties observed in both mouse models when inoculated with most of the animal prion strains tested here (BSE-L, BSE-H and sheep scrapie) suggest that macaque species is an adequate model for the evaluation of human susceptibility to them. However, macaque-PrP is more proficient for classical-BSE prion replication than human-PrP. The use of macaques to model the human condition with regard to classical-BSE infection would take advantage of this property of macaque PrP.

## Methods

### Ethics statement

This study was carried out in accordance with the Directive 2010/63/EU. All protocols were approved by the Committee on the Ethics of Animal Experiments (CEEA) of the Instituto Nacional de Investigación y Tecnología Agraria y Alimentaria (INIA); Permit Number CEEA 2012/002.

### Generation of transgenic mice expressing *Macaca fascicularis* PrP^C^

The transgenic mouse line expressing *Macaca fascicularis* PrP^C^ was obtained as previously described with minor modifications^7^. The open reading frame (ORF) of the macaque PrP gene was isolated by PCR amplification from macaque DNA using primers that created a *Asc*I restriction enzyme site adjacent to the translation start and stop sites (5′-GGCGCGCCATGGCGAACCTTGGCTGCTGGATGCTG-3′ and 5′-GGCGCGCCTCATCCCACTATCAGGAAGATGAG-3′). The PCR fragment was subcloned into vector containing 6.2 kb of the Prp mouse promoter region and the DNA segment from exon I to exon II, which is directly fused to exon III of the Prp gene^44,45^, and the insert was sequenced to confirm no difference in the inferred amino acid sequence with respect to previously sequenced macaque PrP ORF (GenBank accession number NM_001287629). The PrP ORF was excised from the final construct using restriction endonuclease *Not*I and *Sal*I to yield 12.2 kb DNA fragments. The construct was then purified using QIAEX II Gel Extraction Kit (Qiagen). The DNA was resuspended in Tris-EDTA at a final concentration of 2 μg/ml and then microinjected into 237 pronuclear-stage oocytes that were collected from superovulated FVB/N females. The presence of macaque-PrP transgene in the offspring was determined by PCR assay using specific primers for the macaque-PrP ORF: 5′-TTATAGTTGCTGAGCGTCGTCAGGGA-3′ and 5′-TGGGATTCGGTTCCTCCAGGAG-3′ pair and 5′-AGAACAACTTTGTGCACGACTGCGTC-3′ and 5′-CGAAGGAACAAGCAGGAAGGCG-3′ pair.

Seven different lines (founders) of macaque PrP^C^ (macPrP) were obtained. Mouse line with highest levels of macaque PrP^C^ expression was selected for further experiments. Homozygous TgMac mouse line was established backcrossing these animals with homozygous null animals MuPrP^−/−^ ^46^ to obtain a null murine PrP background (PrP mu^−/−^ mac^+/−^). Interbreeding within these animals was performed to obtain homozygosis for the macaque-PrP transgene within a murine PrP background (PrP mu^−/−^ mac^+/+^). The absence of murine PrP gene was determined by PCR using specific primers: 5′-TAGATGTCAAGGACCTTCAGCC-c and 5′-GTTCCACTGATTATGGGTACC-3′.

### Transmission studies

Hu-Tg340 transgenic mice expressing human PrP^C^ (four fold levels than human brain previously described in^16^) and TgMac mice generated here were challenged with a collection of “natural” isolates of distinct origin representative of different TSE agents (see Table 1 for a description of the isolates) as previously described^7^. For that, groups of 5 to 9 individual identified mice (6 to 7 week-old) were inoculated by intra-cerebral route with 20 μl of 10% brain homogenate. As a control, 6 animals of each mouse line were inoculated with healthy cattle brain. All inocula were prepared from brain tissues as 10% (w/v) homogenates in 5% glucose. After inoculation, mice were observed daily and their neurological status assessed twice a week. Animals were killed for ethical reasons when progression of the disease was evident or at the set end point of the experiment, 650 days post inoculation (dpi). Once euthanized, necropsy was performed. A part of the brain was fixed by immersion in 10% buffered formalin for histopathological studies and the other was frozen at −20 °C to determine presence of PrP^res^ by Western blot.

Survival times were calculated as mean ± SD of the dpi of all the mice scored positive for PrP^res^. Attack rate was determined as the ratio of PrP^res^-positive mice among all the inoculated mice.

### Western blot analysis of brain PrP^C^ expression in transgenic mice

PrP^C^ expression was analysed as previously described^7^. Briefly, whole brains from each mouse line were homogenized in extraction buffer (0.5% NP-40, 1% sodium deoxycholate, 10 mM in phosphate-buffered saline pH7.4, with Complete inhibitor cocktail (Roche). Samples were precleared by centrifugation at 2,000 × g for 5 min, after which an equal volume of 2× SDS reducing sample loading buffer was added, and boiled for 5 min before loading onto an 12% Bis-Tris Gel (Criterion XT, BioRad). For immunobloting experiments, the monoclonal antibody 12B2^47^ was used at a concentration of 0.1 µg/mL. 12B2 recognizes _89_-WGQGG-_93_ epitope of the macaque/human PrP^C^ sequence.

Immunocomplexes were detected incubating the membranes for 1 hour with horseradish peroxidase conjugated anti mouse IgG (GE Healthcare Amersham Biosciences). Immunoblots were developed with enhanced chemiluminescence ECL Select (GE Healthcare Amersham Biosciences). Images were captured using ChemiDoc XRS+ System (Bio-Rad) and images were processed using Image Lab 5.2.1 Software (Bio-Rad). All the original Western blot images are presented as Supplementary information.

### Western blot analysis of brain PrP^res^ in transgenic mice

Brain PrP^res^ was analysed as previously described^7,14^. Briefly, 175 ± 20 mg of frozen brain tissue were homogenized in 5% glucose in distilled water in grinding tubes (Bio-Rad) adjusted to 10% (w/v) using a TeSeE^TM^ Precess 48^TM^ homogenizer (Bio-Rad) following manufacturer instructions. Presence of PrP^res^ in transgenic mice brains was determined by Western blot, following the procedure described below and using the reagents of the ELISA commercial test (TeSeE, Bio-Rad). Based on a previously described protocol^48^ 10–100 μl of a 10% (w/v) brain homogenate were treated with proteinase K and the resulting samples were loaded in 12% Bis-Tris Gel (Criterion XT, BioRad). Proteins were transferred electrophoretically onto PVDF membranes (Millipore). For immunoblotting, monoclonal antibodies 12B2 and Sha31^49^ were used at a concentration of 1 µg/mL. Sha31 recognizes _145_-YEDRYYRE-_152_ epitope of the macaque/human PrP^C^ sequence. Immunocomplexes were detected as described above for brain PrP^C^ analysis. N-glycosidase F (PNGaseF, New England Biolabs) was used according to manufacturer’s instructions with minor modifications.

### Histopathology

Mouse brains were analysed as previously described^50,51^ with minor modifications. Briefly, samples were fixed in neutral-buffered 10% formalin (4% formaldehide) before paraffin embedding. After deparaffinization, 4 μm-thick tissue sections were stained with haematoxylin and eosin. Lesion profiles of the brains were established according the standard method described by Fraser and Dickinson^52^. For paraffin-embedded tissue (PET) blots, the protocol described by Andréoletti *et al*.^53^ was used. We conducted PrP^ress^ immunohistochemistry as previously described^51^. Briefly, 4 μm-thick sections were deparaffinized before antigen retrieval. Briefly, sections were immersed in 98% formic acid for 7 min at room temperature and washed in running tap water before being immersed in 4M guanidine isothiocyanate for 1 h at 4 °C. Guanidine isothiocyanate treatment was followed by proteinase K digestion: TBS (50 mM Tris-HCl, 150 mM NaCl, pH 7.6) containing 20 μg/ml of proteinase K for 15 min at 37 °C. Primary antibody incubation was conducted overnight at 4 °C using the Sha31 mAb at 1/1000 dilution. A secondary goat anti-mouse IgG biotinylated antibody (DAKO) diluted 1/200 in 10% normal goat serum was incubated for 30 min at room temperature, and an avidin-biotin-peroxidase complex (Pierce) was applied using diaminobenzidine (DAB) as a substrate. Finally, sections were counterstained with Mayer’s hematoxylin for 1 min, dehydrated, and routinely mounted. Serial sections from positive controls and appropriate negative controls were included in each immunohistochemistry run.

All the procedures described in this section involving prion infected materials were performed within a biosafety level 3 laboratory.

## Supplementary information

Supplemental figures
