# Supplementary material for: Transgenic mouse models expressing human and macaque prion protein exhibit similar prion susceptibility on a strain-dependent manner
Source: Sci Rep. 2019 Oct 30;9:15699. doi: 10.1038/s41598-019-52155-z (PMC6821920; doi:10.1038/s41598-019-52155-z)

Transgenic mouse models expressing human and macaque prion protein exhibit similar prion susceptibility on a strain-dependent manner

Juan Carlos Espinosa[(1)*], Emmanuel E. Comoy[(2)], Alba Marin-Moreno[(1)], Patricia Aguilar-Calvo[(1)], Marie-Christine Birling[(3)], José Luis Pitarch[(1)], Jean-Philippe Deslys[(2)] & Juan María Torres[(1)*]

Figure 2

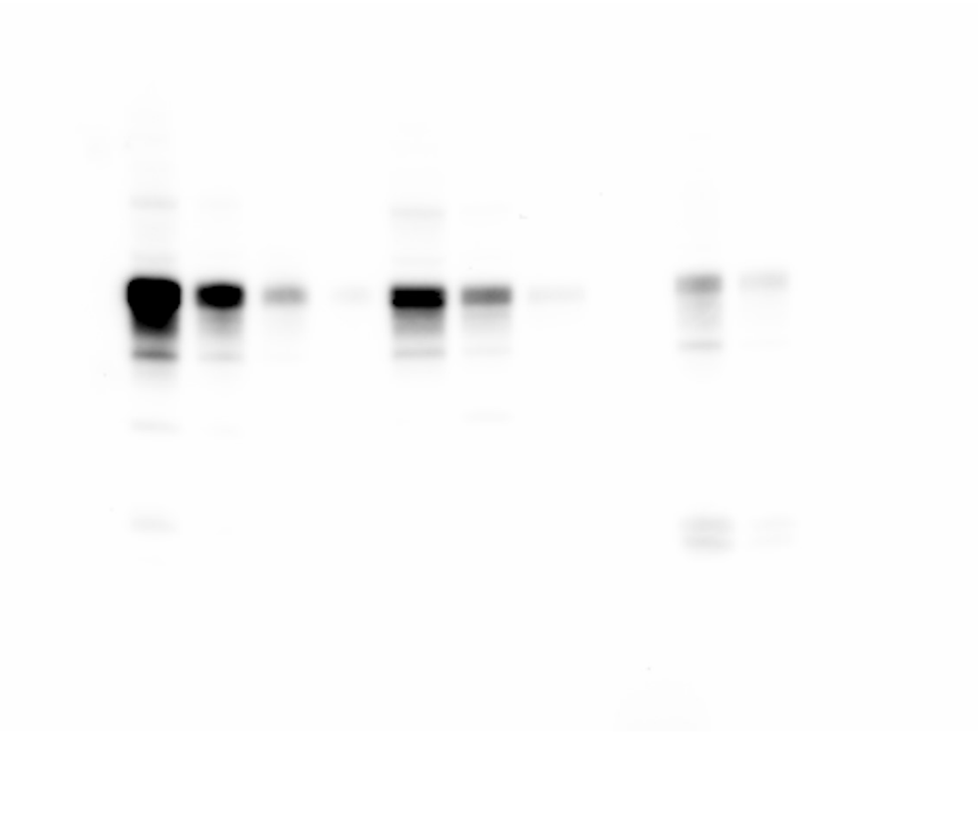

Figure 3

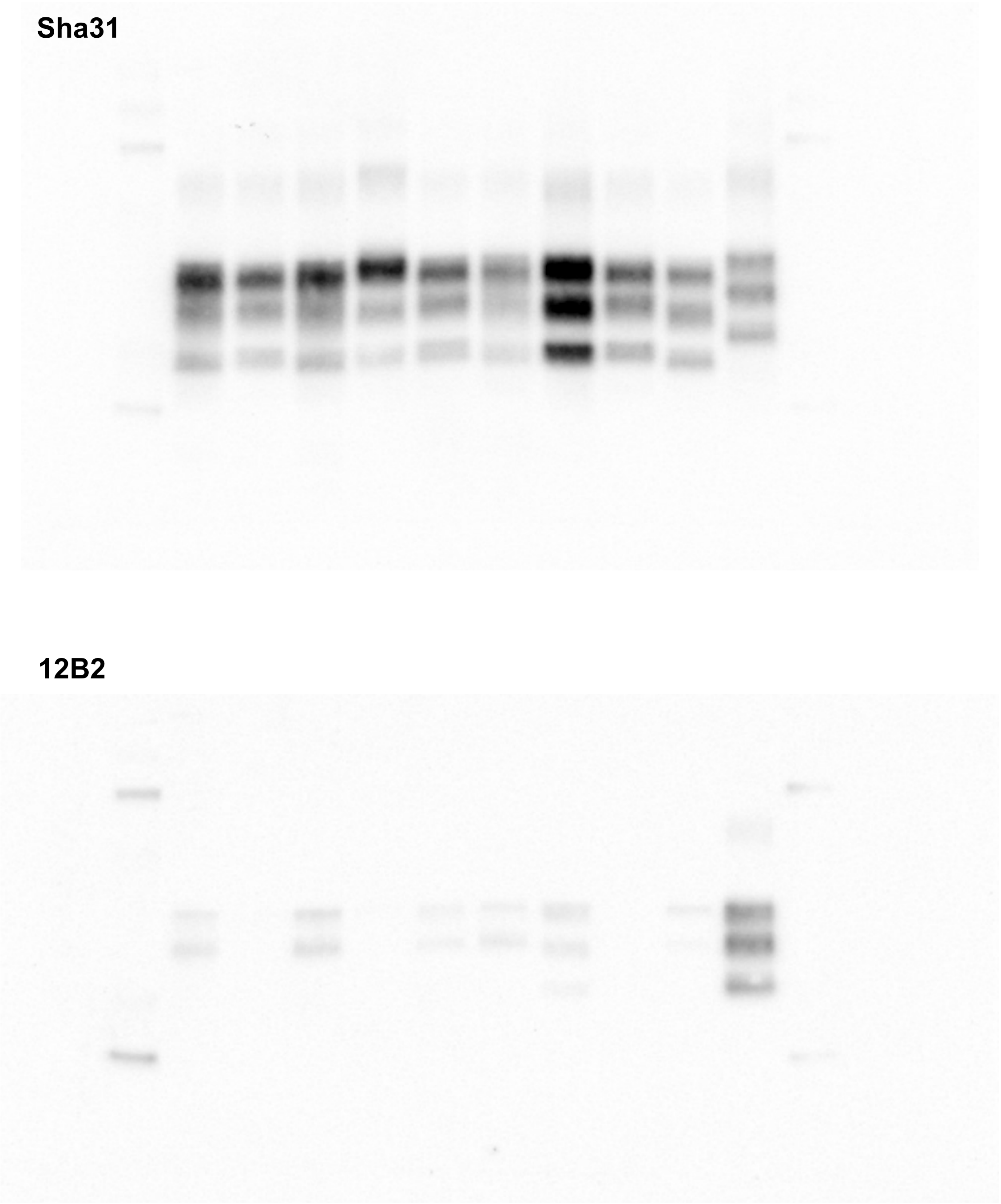

Figure 4

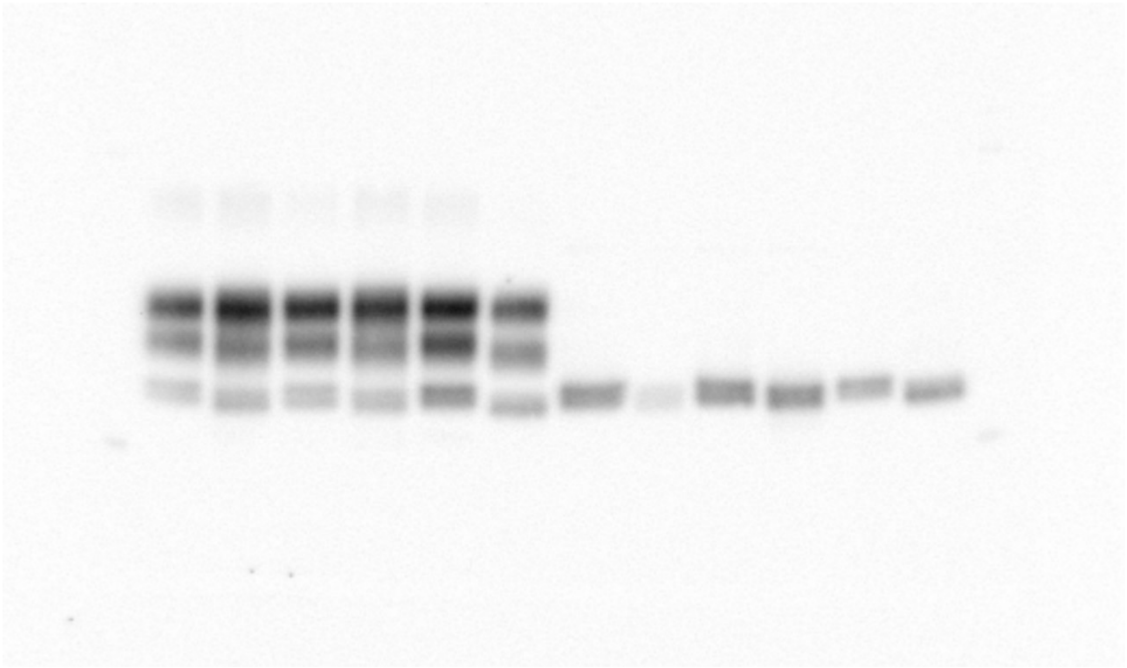

Supplement: Supplementary file 1 — Supplemental figures [file 41598_2019_52155_MOESM1_ESM.pdf]
